# Atypical case of Wolfram syndrome revealed through targeted exome sequencing in a patient with suspected mitochondrial disease

**DOI:** 10.1186/1471-2350-13-3

**Published:** 2012-01-06

**Authors:** Daniel S Lieber, Scott B Vafai, Laura C Horton, Nancy G Slate, Shangtao Liu, Mark L Borowsky, Sarah E Calvo, Jeremy D Schmahmann, Vamsi K Mootha

**Affiliations:** 1 Department of Molecular Biology, Massachusetts General Hospital, Boston, MA 02114, USA; 2 Center for Human Genetic Research, Massachusetts General Hospital, Boston, MA 02114, USA; 3 Department of Systems Biology, Harvard Medical School, Boston, MA 02115, USA; 4 Broad Institute of Harvard and MIT, Cambridge, MA 02141, USA; 5 Diabetes Unit and Endocrine Division, Massachusetts General Hospital, Boston, MA 02114, USA; 6 Ataxia Unit, Cognitive and Behavioral Neurology Unit, Laboratory for Neuroanatomy and Cerebellar Neurobiology, Department of Neurology, Massachusetts General Hospital, Harvard Medical School, Boston MA 02114, USA

## Abstract

**Background:**

Mitochondrial diseases comprise a diverse set of clinical disorders that affect multiple organ systems with varying severity and age of onset. Due to their clinical and genetic heterogeneity, these diseases are difficult to diagnose. We have developed a targeted exome sequencing approach to improve our ability to properly diagnose mitochondrial diseases and apply it here to an individual patient. Our method targets mitochondrial DNA (mtDNA) and the exons of 1,600 nuclear genes involved in mitochondrial biology or Mendelian disorders with multi-system phenotypes, thereby allowing for simultaneous evaluation of multiple disease loci.

**Case Presentation:**

Targeted exome sequencing was performed on a patient initially suspected to have a mitochondrial disorder. The patient presented with diabetes mellitus, diffuse brain atrophy, autonomic neuropathy, optic nerve atrophy, and a severe amnestic syndrome. Further work-up revealed multiple heteroplasmic mtDNA deletions as well as profound thiamine deficiency without a clear nutritional cause. Targeted exome sequencing revealed a homozygous c.1672C > T (p.R558C) missense mutation in exon 8 of *WFS1 *that has previously been reported in a patient with Wolfram syndrome.

**Conclusion:**

This case demonstrates how clinical application of next-generation sequencing technology can enhance the diagnosis of patients suspected to have rare genetic disorders. Furthermore, the finding of unexplained thiamine deficiency in a patient with Wolfram syndrome suggests a potential link between *WFS1 *biology and thiamine metabolism that has implications for the clinical management of Wolfram syndrome patients.

## Background

Mitochondrial diseases are a clinically heterogeneous set of disorders that are caused by defects in mitochondria, the organelles responsible for producing most of the cellular ATP in humans [1]. Multiple organ-systems are typically affected in these disorders, with neurologic and myopathic features being the most prominent [2]. Common clinical features of mitochondrial disease include skeletal myopathy accompanied by exercise intolerance, cardiomyopathy, sensorimotor peripheral polyneuropathy, sensorineural deafness, optic atrophy, diabetes mellitus, seizures, and ataxia [3]. These disorders can be caused by mutations in the nuclear or mitochondrial genomes, and over 100 loci have been identified to date [4].

The clinical and genetic heterogeneity of mitochondrial diseases as well as the technical difficulty of assessing mitochondrial function create a significant diagnostic challenge [5]. One major source of cost and time delays in the clinical evaluation of patients with suspected mitochondrial disease is the use of sequential genetic tests to evaluate individual disorders; as such, a systematic genetic test could accelerate the diagnostic process and reduce overall cost. In the present report, we describe a suspected case of mitochondrial disease whose diagnosis of Wolfram syndrome was ultimately revealed through targeted exome sequencing.

### Case Presentation

In January 2008, a 61-year-old man with a history notable for diabetes mellitus (DM), autonomic neuropathy, diffuse brain atrophy, optic nerve atrophy (OA), and profound amnesia was referred to us to establish neurologic care. The patient carried a diagnosis of multiple system atrophy- cerebellar type (MSAc), principally because of severe cerebellar and brainstem atrophy on MRI.

The patient's early history was remarkable only for childhood bedwetting and urinary urgency as a young adult. He was otherwise well during this time and was a talented athlete who completed college and practiced as an accountant. In his early 20s, he developed bladder dysfunction of unclear etiology requiring intermittent straight catheterization, as well as erectile dysfunction.

At age 33, he was diagnosed with DM, presumed to be type 1, and began treatment with insulin therapy. Although there is no biochemical data available from the time of his original diagnosis, recent testing demonstrated a random C-peptide level of 0.6 ng/mL (reference range 0.9 to 4.3 ng/mL) at a time when his blood glucose was 83 mg/dL. He takes an average of 24 units of insulin per day, and has had good glycemic control with hemoglobin A1c measurements ranging between 6.5 and 7.2% over the last several years. He has had no evidence of retinopathy, or other microvascular or macrovascular complications. He had polyuria and polydipsia at the time of his initial DM diagnosis, but these symptoms resolved once he initiated insulin therapy.

**Figure 1:**
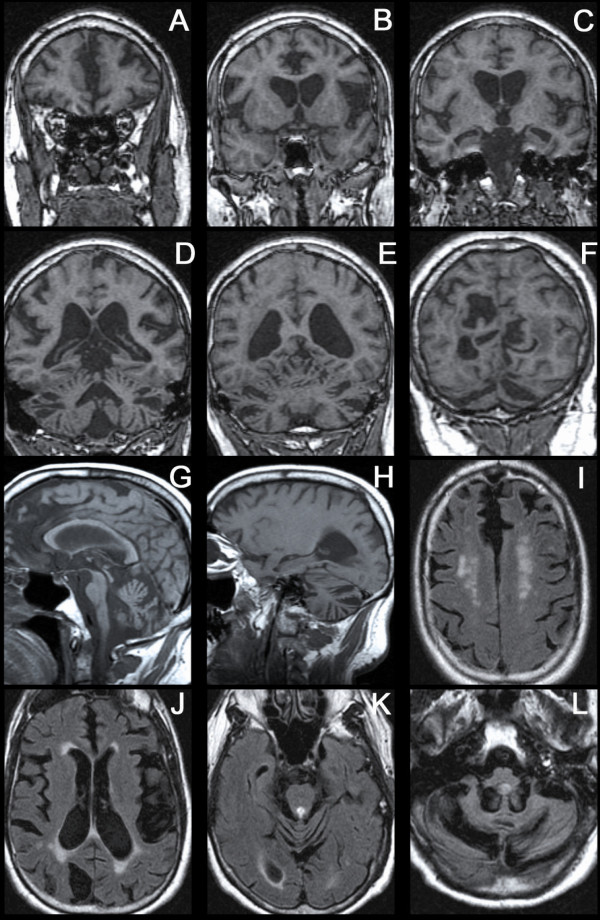
**MRI at age 61 demonstrating severe atrophy of the cerebral hemispheres, cerebellum, and brainstem**. A-F: 3-D SPGR coronal MRI; G, H: T1 sagittal MRI; I-L: FLAIR axial MRI, showing white matter signal hyperintensities.

The patient began dressing in strange colors in his 30s, and color blindness was ultimately diagnosed in his 40s. At age 53, the patient presented for a routine screening ophthalmology exam and was discovered to have bilateral OA with preserved vision. Brain MRI at that time revealed severe atrophy of the cerebellar hemispheres and vermis, pons, and middle cerebellar peduncles as well as moderate cerebral atrophy; a more recent study at age 61 showed these findings as well as more severe cerebral atrophy (Figure 1). Despite these radiographic findings, the patient and his wife reported no gait instability or upper extremity incoordination.

During his late 50s, the patient's neurologic status deteriorated. Formal neuropsychological evaluation revealed profound anterograde amnesia, with additional impairments in cognitive flexibility, executive function, naming, and high order visual processing skills. Attention span, mental tracking, verbal abstract reasoning, complex auditory instructions, and visual spatial functions were preserved. From a psychiatric perspective, he developed symptoms of depression, which responded to treatment with sertraline.

In parallel with the decline in his memory, the patient also developed progressive autonomic neuropathy, with gastroparesis and severe postural hypotension. The autonomic dysfunction exceeded what might be expected from his diabetes mellitus, given his good glycemic control and the absence of other diabetic complications. His bladder dysfunction worsened and he required suprapubic catheter placement at the age of 61. Due to his multiple functional deficits, the patient became unable to work and is now completely reliant upon his wife for care.

Regarding his family history, the patient was born to Ashkenazi Jewish parents and there was no parental consanguinity. His mother died from melanoma, and his father died from multiple strokes and a myocardial infarction. He has two adult daughters, one of whom has attention deficit hyperactivity disorder (ADHD) and Tourette syndrome, while the other suffers from chronic urinary tract infections. His maternal grandmother had type 2 DM, and a maternal first cousin had type 1 DM. No other close relatives have suffered from endocrine, psychiatric, or neurologic disease.

**Figure 2:**
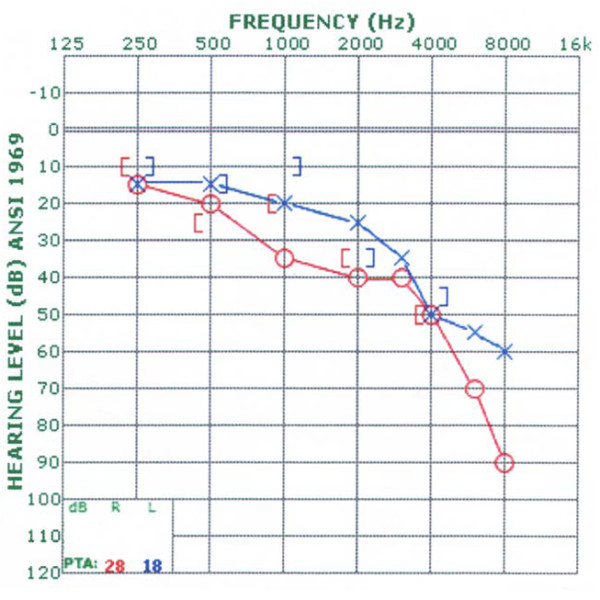
**Results of audiometric testing at age 61 conforming to ANSI 1969 standards**. ANSI = American National Standards Institute; dB = decibel; Hz = hertz; PTA = pure tone average.

On physical examination, he appeared generally medically well. He weighed 79 kilograms and was 178 cm tall, yielding a body mass index of 25. Postural hypotension was evident with systolic blood pressure falling from 150 to 95 after one minute of standing, though asymptomatic. Funduscopic examination revealed optic atrophy bilaterally with no sign of diabetic retinopathy. Visual acuity was 20/40 in each eye. Pupil responses to light and accommodation were normal. Eye movements were normal with the exception of saccadic intrusion into horizontal smooth pursuit. Clinical examination revealed high tone hearing loss bilaterally. Audiometry demonstrated moderate sensorineural hearing loss in the high frequencies on the left, and mild sensorineural hearing loss in the mid-frequencies on the right sloping to a severe loss in the high frequencies (Figure 2). Word recognition was excellent in both ears; 98% on the right and 96% on the left. Muscle tone in the extremities was normal, bulk was intact, and strength was full. There was no evidence of dysmetria with finger-to-nose and heel-to-shin testing, and gait was slow but stable. His affect was flat and he was passive throughout the interview, speaking only when spoken to. He was not oriented to time or place. He could repeat four words, but could not learn them despite multiple attempts. He was unable to provide information concerning major current political or national news. He could, however, recall sizable fragments of remote memory from his college years.

The absence of the cerebellar motor syndrome and the presence of a profound amnestic syndrome on examination called the patient's diagnosis of MSAc into question [6], and we undertook re-evaluation of his case to explore alternate diagnoses. His laboratory work-up revealed an undetectable thiamine level, a surprising finding given his normal diet and the absence of alcohol abuse. We ascribed his amnestic disorder to presumed long-standing thiamine deficiency, but repletion produced minimal clinical impact. The involvement of multiple systems suggested the possibility of a mitochondrial disorder. Genetic testing for OPA1, MELAS, MERFF, LHON and NARP were negative, however analysis of mitochondrial DNA (mtDNA) from a muscle biopsy sample by both Southern blotting and PCR analysis revealed multiple heteroplasmic deletions. Biochemical testing revealed a minor defect in complex I of the electron transport chain. COX and SDH staining of the muscle biopsy specimen were unremarkable, and the mitochondria appeared grossly normal on electron microscopic examination. Occasional central vacuoles and tubular aggregates were seen in the myocytes, which were felt to be consistent with a mild non-specific myopathy.

**Figure 3:**
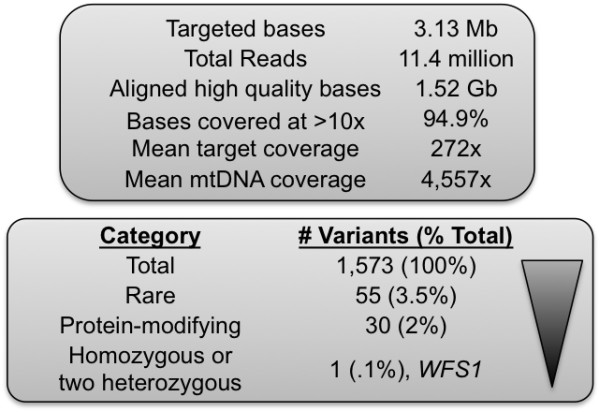
**Results of MitoExome sequencing**. Rare, protein-modifying variants were prioritized, revealing a homozygous c.1672C > T (p.R558C) missense mutation in exon 8 of *WFS1 *that has previously been reported in a patient with Wolfram syndrome [9].

Given the diagnostic uncertainty and concern for a mitochondrial disorder, the patient was enrolled in the mitochondrial disease registry at Massachusetts General Hospital. As part of this program, a sample of the patient's DNA from whole blood underwent targeted exome ("MitoExome") sequencing. Mitochondrial DNA and the exons of 1,600 nuclear genes either encoding mitochondrial proteins or implicated in Mendelian disorders with multi-system phenotypes were targeted using hybrid selection [7]. Amplified targets were sequenced on the Illumina GAIIx platform. Rare, protein-modifying variants found to be homozygous or potentially compound heterozygous were prioritized (Figure 3), revealing an X-linked functional polymorphism c.937G > T (p.D313Y) in *GLA *that is not considered pathogenic [8] and a homozygous c.1672C > T (p.R558C) missense mutation in exon 8 of *WFS1 *that has previously been reported in a patient with Wolfram syndrome [9]. No heteroplasmic mtDNA deletions were detected in whole blood. The patient's *WFS1 *mutation was verified through Sanger sequencing in a CLIA-certified laboratory, though not without complications; the initial report came back negative and only after requested follow-up was the homozygous mutation detected, thereby confirming the diagnosis of Wolfram syndrome.

## Discussion and Conclusions

Wolfram syndrome (OMIM #222300) is an autosomal recessive disorder also known as DIDMOAD for its typical clinical features of Diabetes Insipidus, Diabetes Mellitus, Optic Atrophy and Deafness [10]. The minimum criteria for the diagnosis of Wolfram syndrome are the presence of diabetes mellitus and optic atrophy, both of which usually occur by the age of fifteen [11]. The disorder is also associated with diverse neurologic and psychiatric symptoms, including cognitive impairment [12]. Though with genetic data in hand the diagnosis of Wolfram syndrome now seems clear for the patient presented here, his late age of onset and the prominence of his amnesia are quite atypical for this syndrome and made the diagnosis seem less likely. Furthermore, the clinical presentation, mtDNA deletions, and biochemical data were highly suggestive of a mitochondrial disorder.

Given the multiple organ system dysfunction characteristic of Wolfram syndrome, it was initially thought by a number of clinicians to be a *bona fide *mitochondrial disorder [13]. Further supporting this hypothesis was the observation of mtDNA deletions in some patients with Wolfram syndrome [14-16]. However, in 1998 two groups identified *WFS1*, which encodes an 890 amino acid trans-membrane protein localized to the endoplasmic reticulum, as the gene responsible for the majority of Wolfram syndrome cases, thereby arguing against a mitochondrial etiology [17,18]. Mechanistic studies of *WFS1 *have revealed an important role in dampening the ER stress response in pancreatic beta cells and neurons, and loss of the gene has been shown to result in an exaggerated stress response and accelerated cell death [19]. However, given the shared clinical features between Wolfram syndrome and many mitochondrial disorders, as well as the presence of mtDNA deletions in some patients, many unanswered questions remain about the biology of *WFS1 *and its impact on mitochondrial function and mtDNA maintenance.

Mutations in residue 558 of *WFS1*, as identified in the patient described here, have been previously reported. Our patient's p.R558C mutation was first reported as a heterozygous variant in a cohort of psychiatric patients, though it is unclear whether the variant contributed to disease pathogenesis in that population [20]. Our patient's p.R558C mutation, as well as a p.R558H mutation, have been reported in multiple patients with Wolfram syndrome [9,21,22]. Exome databases suggest that the allele frequency of *WFS1 *c.1672C > T (p.R558C) is quite rare; specifically, the variant is not found in the 1,000 Genomes Project [23] nor in over 2,500 publicly available European and African American exomes [24].

Chaussenot *et al*. specifically discuss the phenotype of a Wolfram case with the same p.R558C mutation identified in our patient in compound heterozygosity with a p.F354del frameshift mutation [12]. Like our patient, Chaussenot *et al*.'s case had significant cerebellar atrophy by MRI, neurogenic bladder symptoms, dementia, DM, OA, as well as the absence of diabetes insipidus. However, unlike our patient, the compound heterozygote case had earlier onset of DM, OA, and neurologic symptoms (at ages 9, 10, and 27 respectively), lacked hearing impairment, and developed cerebellar ataxia and nystagmus as part of her neurologic presentation. Of note, the profound amnestic syndrome seen in our patient was not reported for the compound heterozygote case, and is atypical for Wolfram syndrome patients in general. These cases demonstrate that phenotypic heterogeneity is present even among patients with similar mutations, likely attributable to different genetic and environmental backgrounds.

Some *WFS1 *mutations have been suggested to function in an autosomal dominant manner yielding symptoms such as optic atrophy and hearing impairment in a heterozygous state [25,26]. The patient has one daughter who struggled with Tourette syndrome and ADHD, but the family history is otherwise unremarkable, with no close family members suffering from symptoms associated with Wolfram syndrome. No familial DNA was available to determine the carrier status of his daughter. It is unlikely that his daughter's symptoms are related to *WFS1*, and we suspect that the p.R558C variant likely acts in a recessive manner.

One of the most striking aspects of the case presented here is the profound thiamine deficiency, and its devastating impact on the patient's memory. The symptom complex of diabetes mellitus, deafness, and optic atrophy has been found in association with defects in thiamine metabolism previously in the context of thiamine-responsive megaloblastic anemia syndrome, or Rogers syndrome (OMIM # 249270) [27-29]. Rogers syndrome, a recessive condition caused by mutations in the thiamine transporter *SLC19A2*, is characterized by thiamine-responsive megaloblastic anemia, sensorineural deafness, and DM; its phenotypic similarity to Wolfram syndrome suggests the possibility that disruption of thiamine metabolism may contribute to the pathophysiology of Wolfram syndrome. The coding exons of *SLC19A2 *were sequenced in our patient and no rare mutations were detected, although the presence of non-coding variants cannot be excluded. Given that there was no dietary reason for our patient to develop thiamine deficiency, his case suggests that *WFS1 *may impact thiamine transport or utilization. Furthermore, given that there are no therapies available for the treatment of Wolfram syndrome, assessment of thiamine sufficiency and possibly empiric thiamine supplementation should be considered for these patients.

The patient presented here had a diagnostic journey that is not unusual for disorders involving dysfunction across multiple organ systems, in that several years elapsed and numerous costly genetic tests were performed without a molecular diagnosis. The application of MitoExome sequencing in this case illustrates how clinical application of next-generation sequencing technology can allow for rapid, simultaneous evaluation for multiple genetic disorders with a single test thereby accelerating the diagnostic process. Furthermore, this case demonstrates how careful study of an individual patient with a rare disorder can yield potentially new insight into gene function and disease pathogenesis.

## Consent

Written informed consent was obtained from the patient and his wife for publication of this case report.

## List of abbreviations

mtDNA: mitochondrial DNA; MRI: Magnetic Resonance Imaging; MSAc: multiple system atrophy, cerebellar-type; DM: diabetes mellitus; DI: diabetes insipidus; OA: optic atrophy; ADHD: attention deficit hyperactivity disorder.

## Competing interests

The authors declare that they have no competing interests.

## Authors' contributions

SBV & JDS looked after the patient. DSL, NGS, SL, MLB, & SEC generated and analyzed the sequence data. DSL, SBV, LCH, JDS, & VKM wrote the report. All authors read and approved the final manuscript

## Pre-publication history

The pre-publication history for this paper can be accessed here:

http://www.biomedcentral.com/1471-2350/13/3/prepub
